# Cholesterol Enhances the Toxic Effect of Ethanol and Acetaldehyde in Primary Mouse Hepatocytes

**DOI:** 10.1155/2016/9209825

**Published:** 2015-12-14

**Authors:** Anayelly López-Islas, Victoria Chagoya-Hazas, Benjamin Pérez-Aguilar, Mayrel Palestino-Domínguez, Verónica Souza, Roxana U. Miranda, Leticia Bucio, Luis Enrique Gómez-Quiroz, María-Concepción Gutiérrez-Ruiz

**Affiliations:** ^1^Departamento de Ciencias de la Salud, Universidad Autónoma Metropolitana Iztapalapa, 09340 México, DF, Mexico; ^2^Instituto de Fisiología Celular, Universidad Nacional Autónoma de México, 04510 México, DF, Mexico; ^3^Red Fisiopatología de las Enfermedades Hepáticas, PRODEP-SEP, México, DF, Mexico

## Abstract

Obesity and alcohol consumption are risk factors for hepatic steatosis, and both commonly coexist. Our objective was to evaluate the effect of ethanol and acetaldehyde on primary hepatocytes obtained from mice fed for two days with a high cholesterol (HC) diet. HC hepatocytes increased lipid and cholesterol content. HC diet sensitized hepatocytes to the toxic effect of ethanol and acetaldehyde. Cyp2E1 content increased with HC diet, as well as in those treated with ethanol or acetaldehyde, while the activity of this enzyme determined in microsomes increased in the HC and in all ethanol treated hepatocytes, HC and CW. Oxidized proteins were increased in the HC cultures treated or not with the toxins. Transmission electron microscopy showed endoplasmic reticulum (ER) stress and megamitochondria in hepatocytes treated with ethanol as in HC and the ethanol HC treated hepatocytes. ER stress determined by PERK content was increased in ethanol treated hepatocytes from HC mice and CW. Nuclear translocation of ATF6 was observed in HC hepatocytes treated with ethanol, results that indicate that lipids overload and ethanol treatment favor ER stress. Oxidative stress, ER stress, and mitochondrial damage underlie potential mechanisms for increased damage in steatotic hepatocyte treated with ethanol.

## 1. Introduction

Nonalcoholic fatty liver disease (NAFLD) and alcoholic liver disease (ALD) are the most common life-style liver diseases caused by obesity and alcohol intake, respectively [1, 2]. Epidemiological evidence shows that obese alcoholics have 2-3 times higher risk of developing steatohepatitis or cirrhosis as compared to nonobese alcoholics or obese nonalcoholics [3]. Obesity and alcohol consumption have some common mechanisms that may contribute to exacerbation of liver damage when these conditions coexit, including oxidative stress, cytochrome P450 (Cyp)2E1 induction, increased lipid synthesis, free fatty acids, and endoplasmic reticulum (ER) stress [4].

Epidemiological data connect an increase in cholesterol intake to the risk and severity of NAFLD and emerging experimental and human data suggest that disturbed hepatic cholesterol homeostasis and free cholesterol accumulation are relevant to the pathogenesis of NAFLD [5]. Free cholesterol accumulation damages hepatocytes by disturbing mitochondrial and ER membrane integrity, generating mitochondrial oxidative injury and ER stress [5]. ER controls cellular cholesterol levels through pathways that sense the level of cholesterol or cholesterol derivatives within the ER membrane itself. Hepatocyte lipid accumulation induces Cyp2E1, and the increase activity of Cyp2E1 in steatosis leads to tissue oxidative stress and production of reactive oxygen species.

Recent data from our lab show that lipid overloaded in VL-17A cells makes them more susceptible to cell damage and oxidative stress when treated with ethanol [6].

As mechanisms underlying synergistic hepatocyte injury caused by cholesterol and ethanol are not clear, our aim in this study was to evaluate the effect of ethanol or its metabolite, acetaldehyde, on hepatocytes primary culture obtained from mice fed for two days with a high cholesterol diet determining the viability, oxidative stress, content and activity of Cyp2E1, and ER stress.

## 2. Materials and Methods

### 2.1. Mice, Hepatocytes Isolation, and Culture

Eight- to ten-week-old male C57BL/6 mice were maintained in pathogen-free housing and cared for in accordance with the Universidad Autonoma Metropolitana Guide for the Care and Use of Laboratory Animals.

Mice were fed with a high cholesterol diet (HC, 2% cholesterol + 0.5% of sodium cholate) for two days. Control mice were fed with regular standard chow (CW) rodent diet. After the two days under diets, mice were subjected to the two-step method of collagenase perfusion for hepatocyte isolation as described previously [7]. The viability was >90% as assessed by trypan blue exclusion. Primary hepatocytes from both HC and chow diet fed mice were seeded at 2.13 × 10^5^ cells per cm^2^ in 10 cm dishes (Nalge Nunc) in Ham's F-12/Dulbecco's modified Eagle's basal hepatocyte growth medium supplemented with 10% fetal bovine serum (Hyclone Lab Inc., Logan, UT), 1% antibiotics in a humidified atmosphere of 5% CO_2_/95% air. All experiments were carried out using 225,000 cells/cm^2^ seeded in 10 cm dishes in at least three independent experiments carried out in triplicate.

### 2.2. Treatments

After six h stabilization, culture media were exchanged for one without serum and containing ethanol (Et) 100 mM or acetaldehyde (Ac) 200 *μ*M; both treatments lasted 24 h. In order to lessen Et and Ac evaporation, all dishes were wrapped with parafilm during time of treatment.

### 2.3. Oil Red O Staining

Cell lipid content was determined by oil red O staining as previously reported [6]. Briefly, cells were fixed in 4% formalin and stained with oil red O solution (0.35% in 60% isopropanol) for 10 min. Cells were counterstaining with hematoxylin.

The intracellular lipid droplets were stained by oil red O; the cell cultures were washed with PBS pH 7.4 and fixed for 20 min with 4% formalin in 0.05 M PBS; after washing with sterile double distilled water and 60% isopropanol for 2 min, the cells were stained with 0.35% oil red O solution in 60% isopropanol for 10 min at room temperature.

### 2.4. Cell Viability by Crystal Violet

Cell viability was determined by crystal violet staining method as we previously reported [8].

### 2.5. Transmission Electron Microscopy (TEM)

After washing with PBS, samples were fixed with 2.5% glutaraldehyde plus paraformaldehyde in PBS (pH 7.4) for 2 h and washed three times for 30 min in PBS. After that, glutaraldehyde-fixed specimens were treated with 1% OsO_4_ in PBS for 2 h, dehydrated in increasing concentrations of ethanol (50%–100%), infiltrated with propylene oxide, and embedded in an EPON mixture. Polymerized sections were then cut, stained, and examined using transmission electron microscopy (JEOL JEM 12000 EII).

### 2.6. Western Blot Analysis

Total protein was isolated from cells, with M-Per tissue protein extraction reagent (Pierce, Rockford, IL, USA), containing 1% halt protease inhibitor mixture (Pierce), 100 mM sodium fluoride, 1 mM phenylmethylsulfonyl fluoride (PMSF), and 50 mM sodium orthovanadate. One hundred *μ*g of total protein was separated on NuPAGE novex 4–20% gels (Invitrogen), transferred to polyvinylidene difluoride membranes (Invitrogen), and probed with anti-Cyp2E1 (Cell Signaling, Inc.) or anti-PERK (Abcam Inc.), followed by incubation with the specific horseradish peroxidase-conjugated secondary antibody (GE Healthcare, Inc., Uppsala, Sweden). Immunoreactive bands were identified with ECL-Plus Western blotting detection reagent (GE Healthcare). Equal loading was demonstrated by probing the membrane with anti-actin.

### 2.7. Immunofluorescence and Filipin Staining

Immunofluorescence was carried out as we previously published [9]. Briefly, hepatocytes were fixed with 4% paraformaldehyde for 15 min. Coverslips were blocked using BSA 5% + triton 0.3% for 30 min at room temperature. Cells were incubated with a mouse anti-ATF6 monoclonal antibody (10 *μ*g/mL, Abcam) overnight at 4°C, followed by incubation with a secondary anti-mouse IgG antibody, conjugated to Alexa Fluor 647 (Invitrogen) for 1 h at 25°C. DAPI was used to stain the cell nuclei.

Free cholesterol was identified by confocal microscopy using filipin (250 *μ*g/mL, Sigma-Aldrich) as reported by Marí and coworkers [10]. Microphotography was recorded in a Carl Zeiss NLO 780 confocal microscopy.

### 2.8. Microsomal Fractions

Cell cultures were washed with PSB 24 h after treatment with Et or Ac. The cell pellet was resuspended vigorously using an insulin syringe for 5 cycles (4 times per cycle) in 600 *μ*L of lysis buffer (20 mM HEPES, 2 mM KCl, 2 mM MgCl_2_, 250 mM sucrose, and an inhibitor cocktail protease) to pH 7.4. Cells were always maintained on ice during this procedure. The suspension was centrifuged at 1,000 g for 5 min at 4°C. The supernatant was recovered and centrifuged at 8000 g for 10 min at 4°C. The resulting supernatant was transferred to ultracentrifuge tubes (Beckman Optima TLX) and centrifuged at 100,000 g for 1 h at 4°C. The supernatant was discarded and the pellet was resuspended in 30 *μ*L of storage buffer (it contains the same lysis buffer without sucrose).

### 2.9. Cyp2E1 Activity Assay

Cyp2E1 activity was assayed by the nitrocatechol method. 10 *μ*L of* p*-nitrophenol (5 mM) and 25 *μ*L of NADPH (20 mM) were added to 440 *μ*L of assay buffer (50 mM potassium phosphate) to pH 7.4 and were incubated at 37°C for 15 min. After that, 100 *μ*g of microsomal protein was added and the reaction was left for 1 h at 37°C. To stop the reaction, 100 *μ*L of trichloroacetic acid was added (20%). The samples were placed on ice for 2 min. The samples were centrifuged at 10,000 g for 5 min. 500 *μ*L of the supernatant obtained was transferred to Eppendorf tubes and we added 250 *μ*L of NaOH (2 M). The mixture was agitated with vortex and read at 535 nm in a multiplate reader spectrophotometer (Beckman DU640).

To determine the enzymatic activity, the molar extinction coefficient of the formed product (nitrocatechol, 9.53 × 10^5^ M^−1^ CM^−1^) was considered, as well as the absorbance of each sample and the reaction time. Results were expressed as nmol of product per hour per microgram of microsomal protein.

### 2.10. Protein Oxidation

Carbonyl modification of proteins, a key biomarker for the identification of oxidative stress, was addressed by using Oxyblot Protein Oxidation Detection Kit (Millipore, Darmstadt, Germany).

### 2.11. Protein Content

The protein content was determined by using the bicinchoninic acid method (BCA kit, Pierce Inc.), following manufacturer's instructions.

### 2.12. Statistical Analysis

The data are presented as mean ± SEM for at least three independent experiments carried out in triplicate. Comparisons between groups were performed by one-way analysis of variance (ANOVA) with Bonferroni post hoc test using GraphPad Prism 5.0 for Mac OS X. Differences were considered significant at ^*∗*^ *p* ≤ 0.05.

## 3. Results

### 3.1. A HC Diet Induces Lipid Overload in Mice Hepatocytes and Sensitizes to Alcohol and Acetaldehyde Damage

**Figure 1:**
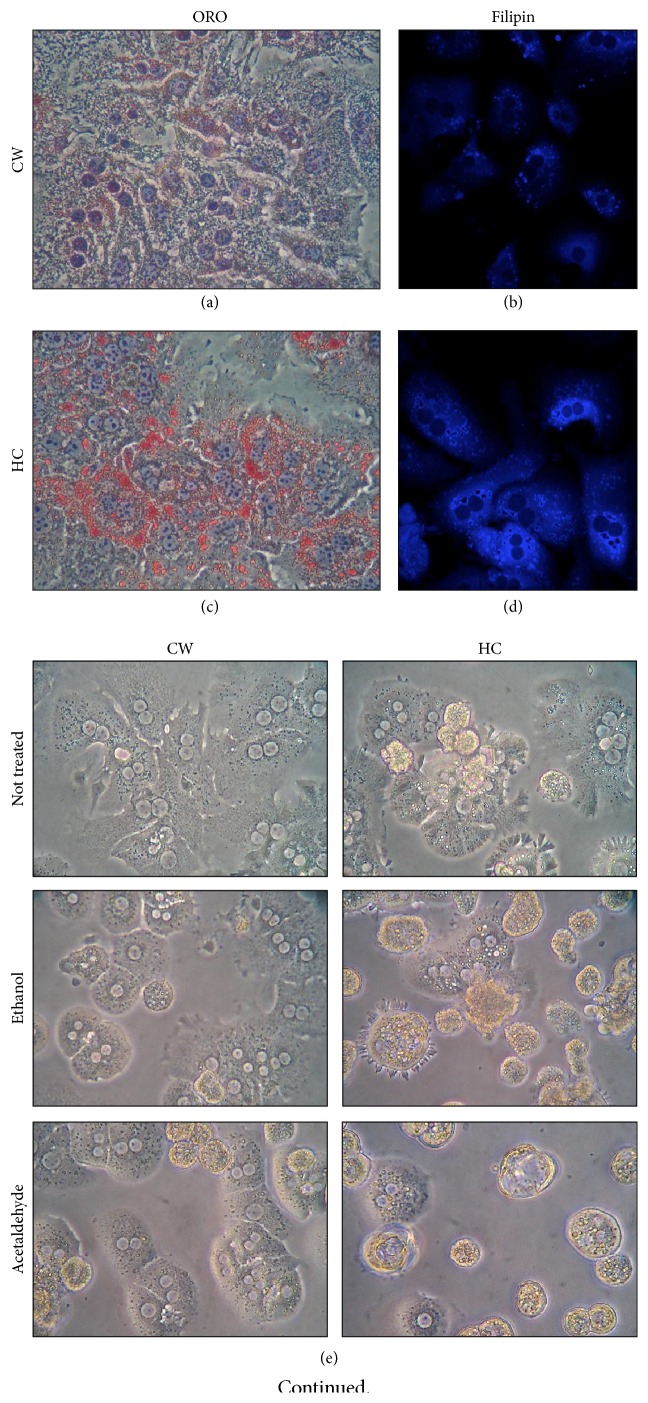
A high cholesterol diet induces neutral lipids and free cholesterol overload in mouse hepatocytes and decreases cell viability under ethanol and acetaldehyde treatments. Mice were fed with a high cholesterol diet (HC, 2% cholesterol) or regular standard chow (CW) for two days. Hepatocytes were isolated and plated. Oil red staining (ORO) in CW cells (a) and HC cells (c) for neutral lipids identification and filipin staining in CW cells (b) and HC cells (d) for free cholesterol determination were assayed. (e) Cell morphology determined by bright field microscopy of CW and HC cells under ethanol (100 mM) and acetaldehyde (200 *μ*M) treatments. Cell viability assessed by crystal violet staining in (f) CW and (g) HC cells treated with ethanol (Et) and acetaldehyde (Ac). Each bar represents mean ± SEM of three independent experiments. Differences were considered significant at ^*∗*^ *p* ≤ 0.05 versus NT cells. Images are representative of at least three independent experiments. Original magnification 320x.

Mice fed with a HC or regular CW diet for two days were subjected to liver perfusion for the isolation of hepatocytes. After 6 h of seeding, and prior to treatments, HC cells clearly exhibited an overload of neutral lipids and free cholesterol, judged by ORO and filipin staining (Figure 1(a)), confirming the steatotic effect of the HC diet; these data were in agreement with our previous work in which we reported 3- and 2.2-fold increase of cholesterol and neutral lipids, respectively, in HC cells compared with CW [11].

HC and control CW cells were treated with Et (100 mM) or Ac (200 *μ*M) for 24 h; HC cellular morphology was clearly affected in both treatments, observing that detached death cells were present in culture dishes, and many stressed cells remained adhered as depicted in Figure 1(e). To gain confidence cell viability was assayed by using crystal violet staining; Figure 1(g) shows that HC diet sensitized cells to the toxic effect of Et and Ac by decreasing cell viability; however no significant effect was observed in CW cells (Figure 1(f)).

### 3.2. HC Diet Increases Cyp2E1 Expression and Activity Inducing Oxidative Stress

**Figure 2:**
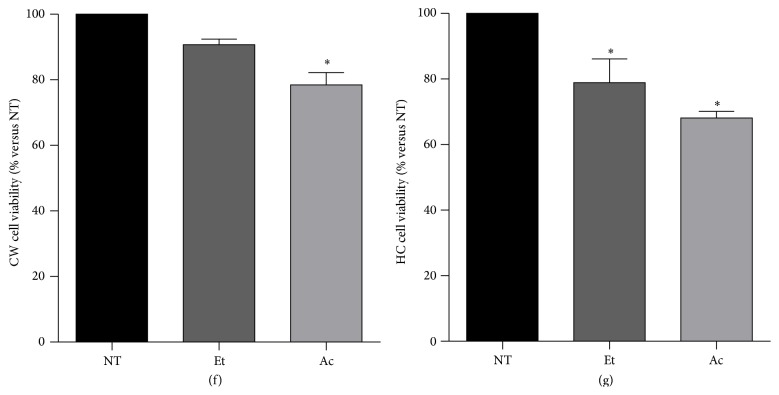
High cholesterol diet induces both Cyp2E1 overexpression and activity. Whole cell lysate was obtained from CW and HC cells treated or not with ethanol (Et, 100 mM) or acetaldehyde (Ac, 200 *μ*M) and subjected to Western blotting. (a) Representative image of the Cyp2E1 immunoblot. (b) Densitometric analysis of protein content relative to actin used as loading control. (c) Cyp2E1 activity. (d) Protein oxidation determined by Oxyblot kit. Each column represents mean ± SEM of at least four independent experiments. Differences were considered significant at ^*∗*^ *p* ≤ 0.05 versus NT cells. Images are representative of at least four independent experiments.

In order to find a mechanism to explain the sensitization, we were focused to address the status of Cyp2E1, the main Et metabolism system with clearly toxic consequences [8]. Interestingly, HC diet promoted the overexpression of Cyp2E1 as Western blot result displayed (Figures 2(b)-2(c)), with no significant changes with Et and Ac treatments. CW cells did not present changes in Cyp2E1 protein content. We proceeded to study the Cyp2E1 activity in microsomes obtained from CW and HC cells treated with both toxics.  Figure 2(c) shows a significant increment in the activity in HC nontreated cells compared with CW cells; Et treatment did not modify the activity; however, remarkably, Ac treatment considerably decreased the enzyme activity. Regarding CW cells, only Et induced an increment as expected.

Elevation in Cyp2E1 activity is directly associated with oxidative damage as we previously reported [8], and in order to confirm this phenomenon we performed an oxyblot to address the protein oxidation content; HC diet induced protein oxidation, with no significant differences in Et and Ac treatments. A slight increment was observed in CW cells treated with Et compared with CW nontreated cells; data are in agreement with Cyp2E1 activity (Figure 2(d)).

### 3.3. HC Diet Induces ER Stress

**Figure 3:**
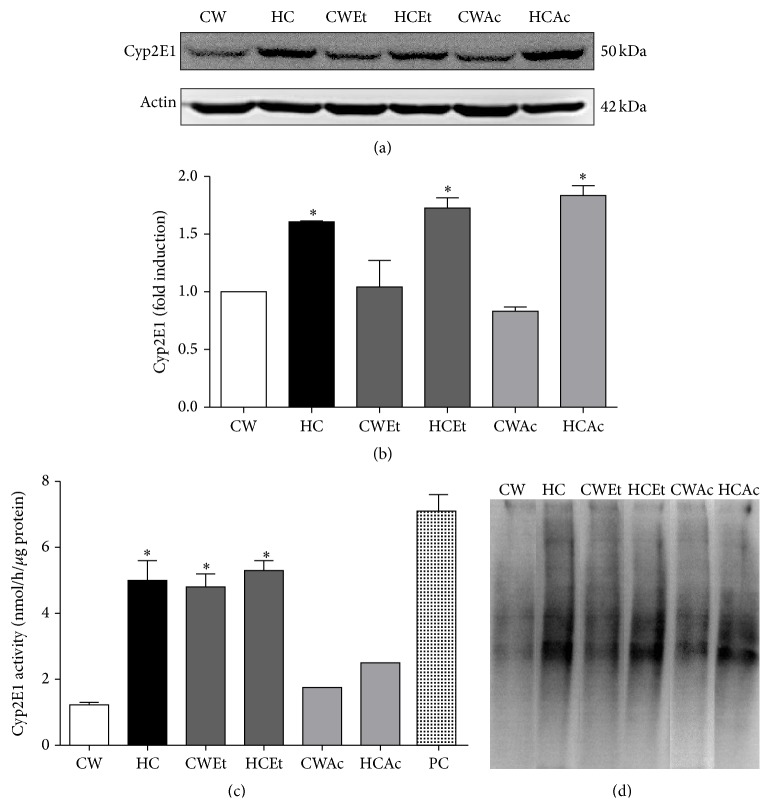
Cholesterol overload induces cell morphology changes. Cell morphology determined by transmission electron microscopy of CW and HC cells under ethanol (100 mM) and acetaldehyde (200 *μ*M) treatments. M: mitochondria; ER: endoplasmic reticulum; *∗*: mitochondria fission; black arrow: autophagosome. Bar 200 nm. Images are representative of at least three independent experiments. (g) Microsomal cholesterol determination. Each column represents mean ± SEM of at least four independent experiments. Differences were considered significant at ^*∗*^ *p* ≤ 0.05 versus CW cells.

It is known that disturbances in lipid homeostasis induce cellular stress, particularly at mitochondria and endoplasmic reticulum level [10, 12]. An analysis by TEM pointed out that ER was clearly compromised in HC cells, presenting dramatic loss in ER architecture (Figure 3(d)) in comparison with CW cells (Figure 3(a)), seeing that Et treatment induced megamitochondria formation (Figures 3(b) and 3(e)), as we previously reported [13]. Interestingly we observed autophagosomes formation only in HC cells treated with Et (arrow, Figure 3(e)), suggesting that autophagy could play a prominent role. Ac treatment alone did not induce significant changes in the ER architecture, but we observed megamitochondria and some structures that suggest mitochondrial fission (Figure 3(f), asterisk). In order to support the idea that ER overloaded cholesterol, we assayed biochemically its content in microsomes isolated from CW and HC cells, confirming that certainly ER in HC cells presented an increment in cholesterol content.

**Figure 4:**
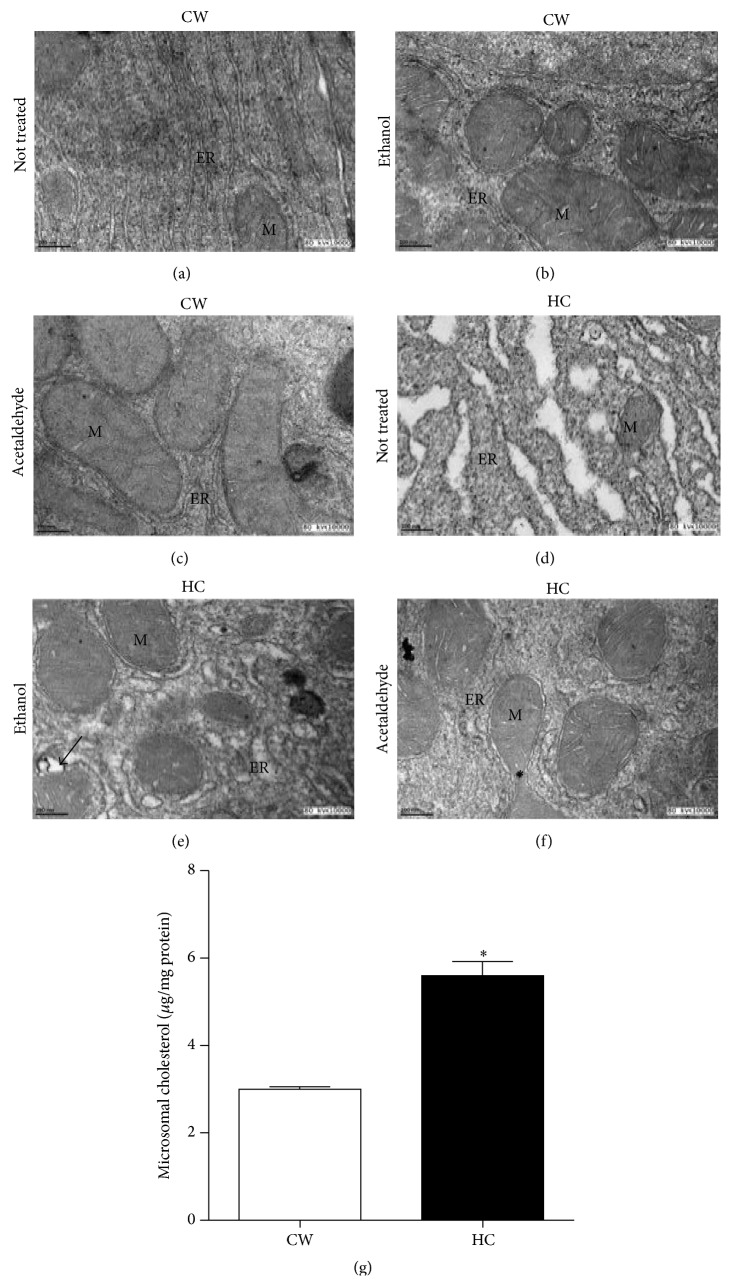
Cholesterol induces endoplasmic reticulum stress. Whole cell lysate was obtained from CW and HC cells treated or not with ethanol (Et, 100 mM) or acetaldehyde (Ac, 200 *μ*M) and subjected to Western blotting. (a) Representative image of PERK immunoblot. (b) Densitometric analysis of protein content relative to actin used as loading control. (c–h) ATF6 nuclear translocation determined by confocal microscopy. ATF6 is in red and nucleus in blue. Images are representative of at least four independent experiments. Original magnification 320x.

Finally, to further address the ER stress we measure the content of PERK, a key regulator of the process. We found that HC diet exhibited an increment in the protein, effect observed in Et treatment in both CW and HC cells; interestingly, Ac did not exhibit any effect in PERK (Figures 4(a)-4(b)). Nuclear translocation of ATF6 is another key marker of ER stress; we identified nuclear ATF6 positive signal only in HC cells treated with Et, coinciding with less fluorescence signal in the cytosol (Figure 4(g)). The result supports the idea that cholesterol overload and Et treatment favor ER stress.

## 4. Discussion

Alcohol intake is one of the most important risk factors for liver disease [1], in addition to hepatitis B and hepatitis C virus infection; however liver lipid overload has been positioned as a clear risk factor for liver disease. We recently reported that lipid overload, predominantly cholesterol, induces oxidative stress and cell damage [14], and this could be a key determinant for initiation and progression of the disease, particularly when the steatotic phenotype is accompanying another risk factor such as alcohol. Previously, we reported that cells under a free fatty acids overload are more susceptible to alcohol metabolism damage [6]. In the present work we focused on addressing the hepatic cell damage under high cholesterol content and the presence of Et and its toxic metabolite—Ac.

Our data show that HC cells are more susceptible to the toxic effects of Et and Ac, inducing important changes in cellular morphology, with no apparent effects in control CW cells. It is well known that oxidative stress induced by alcohol biotransformation is the main mechanism of toxicity, mainly by the activity of the Cyp2E1, as we previously probed [8]. However, ethanol metabolism toxicity reached significant differences at 48 h after treatment in cells that overexpress both Cyp2E1 and alcohol dehydrogenase (VL-17A) [8, 15]. Interestingly, in primary hepatocytes culture with high cholesterol content, we observed the toxic effects of both Et and Ac, at 24 h (Figures 1(e)–1(g)), suggesting that cholesterol could be inducing the expression or activity of the Cyp2E1 system.

It is well known that Cyp2E1 is also related to fatty acids biotransformation and it is positioned as a possible risk factor for the severity of NAFLD [16]. Our results indicate that the diet alone increased both expression and activity of Cyp2E1, possibly as an adaptive response; however the metabolism of fatty acids by this enzyme complex is related to the production of toxic intermediaries that could increase the damage [16]. Results depicted in Figure 2 clearly show that HC diet by itself increased both Cyp2E1 protein content and activity, which remained increased with both treatments. In CW cells did not increase the content, but Et induced a rise in the enzyme activity as expected; these results were in agreement with protein oxidation.

Changes in ER observed by TEM revealed significant changes in the structure of the cisternae, indicating ER stress. To confirm that ER is overloading cholesterol, we assayed the content of this lipid. The result indicated 1.8-fold the values of the CW cells. We observed megamitochondria formation in Et treatments, an undoubted effect due to oxidative stress and due also to heavy metals [17] and alcohol toxicity [18, 19]. Also, we detected autophagosomes in Et treated HC cells, but not in other treatments (Figure 3(e), arrow), indicating severe damage that is in agreement with other studies that pinpoint this effect in extensive injury, particularly by alcohol and hepatitis C virus infection [20]. This effect could serve as a protective mechanism in order to restore the normal function of the cell by eliminating compromised organelles such as mitochondria or misfolded proteins [21, 22].

Interestingly, Ac treatment induced mitochondrial fission, judged by TEM (Figure 3(f), asterisk) with modest changes in ER; although it is reported that Ac induces ER stress in HepG2 cells [23], we could not find significant changes in TEM studies.

Finally, the analysis of key markers of ER stress revealed that HC diet induced the expression of PERK, one of the sensors in ER stress that phosphorylates downstream substrates such as elF2*α*, which, in addition, impedes protein synthesis [24]. Et treatment sustained this increment, but remarkably Ac did not affect PERK expression. Ethanol by itself induced the elevation of PERK. The analysis of ATF6 nuclear localization showed positive signal only in ethanol treated HC cells, and once again we could not find changes in Ac treated cells. Data suggest that Ac induces predominantly damage by a mechanism dependent on mitochondria [10, 23] in presence and absence of cholesterol overload, but our results clearly show that cholesterol overload sensitizes cells to ethanol toxicity.

In conclusion, cholesterol overload is a determinant factor that could maintain or aggravate the alcohol-induced liver damage; a close surveillance of lipid overload is desired, particularly cholesterol, in the liver of those patients with alcohol abuse in order to avoid the exacerbation of the disease. Alcohol withdrawal is fundamental for the treatment of liver related disorders in obese patients.
